# Coeliac Patients Are Undiagnosed at Routine Upper Endoscopy

**DOI:** 10.1371/journal.pone.0090552

**Published:** 2014-03-04

**Authors:** Kathryn Robson, Michelle Alizart, Jarad Martin, Robyn Nagel

**Affiliations:** 1 Toowoomba Gastroenterology Clinic, Medici Medical Centre, Toowoomba, Queensland, Australia; 2 Radiation Oncology Queensland, St Andrew’s Hospital, Toowoomba, Queensland, Australia; 3 Rural School of Medicine, University of Queensland, Toowoomba, Queensland, Australia; University Hospital Llandough, United Kingdom

## Abstract

**Background and Aims:**

Two out of three patients with Coeliac Disease (CD) in Australia are undiagnosed. This prospective clinical audit aimed to determine how many CD patients would be undiagnosed if duodenal biopsy had only been performed if the mucosa looked abnormal or the patient presented with typical CD symptoms.

**Methods:**

All eligible patients presenting for upper gastrointestinal endoscopy (OGD) in a regional center from 2004–2009 underwent prospective analysis of presenting symptoms and duodenal biopsy. Clinical presentations were defined as either Major (diarrhea, weight loss, iron deficiency, CD family history or positive celiac antibodies- Ab) or Minor Clinical Indicators (CI) to duodenal biopsy (atypical symptoms). Newly diagnosed CD patients had follow up celiac antibody testing.

**Results:**

Thirty-five (1.4%) new cases of CD were identified in the 2,559 patients biopsied at upper endoscopy. Almost a quarter (23%) of cases presented with atypical symptoms. There was an inverse relationship between presentation with Major CI’s and increasing age (<16, 16–59 and >60: 100%, 81% and 50% respectively, p = 0.03); 28% of newly diagnosed CD patients were aged over 60 years. Endoscopic appearance was a useful diagnostic tool in only 51% (18/35) of CD patients. Coeliac antibodies were positive in 34/35 CD patients (sensitivity 97%).

**Conclusions:**

Almost one quarter of new cases of CD presented with atypical symptoms and half of the new cases had unremarkable duodenal mucosa. At least 10% of new cases of celiac disease are likely to be undiagnosed at routine upper endoscopy, particularly patients over 60 years who more commonly present atypically. All new CD patients could be identified in this study by performing pre-operative celiac antibody testing on all patients presenting for OGD and proceeding to biopsy only positive antibody patients and those presenting with either Major CI or abnormal duodenal mucosa for an estimated cost of AUS$4,629 and AUS$3,710 respectively.

## Introduction

Coeliac Disease (CD) is an immune-mediated disorder of the small bowel affecting 0.5–1% of the Australian population [1]–[8]. Exposure of genetically susceptible individuals to gluten leads to inappropriate activation of the body’s immune system [3]–[4] resulting in the production of antibodies (Ab) to gluten as well as against some of the body’s own tissues including endomysium and tissue transglutaminase. This subsequent immune response results in small-bowel mucosal inflammation and the various degrees of villous atrophy that are microscopically characteristic of CD [1], [4]–[6].

CD has a highly protean clinical presentation and has been described as ‘The New Great Imitator’ [3]. Three clinical variations in presentation have been described: the typical (obvious gastrointestinal symptoms: steatorrhoea, diarrhoea, weight loss, and failure to thrive [1], [5]–[10]); the atypical or subclinical (presenting with largely non- gastrointestinal or non-specific gastrointestinal symptoms: [1], [3], [6], [7], [10]); and the asymptomatic (silent) forms [9], [11], [12]. Difficulties arise diagnosing the CD patients presenting with no clinical suspicion of CD or subtle mucosal changes and these patients are likely to remain undiagnosed for decades (as many as 7 out of 8 patients with CD may remain undiagnosed [11]). There are benefits of reduced morbidity and mortality in diagnosing CD in a prompt manner [5], [10], [13], [14].

We conducted a prospective clinical audit of all patients presenting for an upper endoscopy (OGD) over a 5-year period, all of which had had a duodenal biopsy included as part of the clinical evaluation, to determine what investigative strategy would most accurately diagnose all cases of CD. We were interested to determine which clinical symptoms are most relevant in predicting the diagnosis of CD, whether age or gender had any effect on presentation and if we could improve the use of healthcare resources.

## Materials and Methods

### Patients

This is an audit of all newly diagnosed CD patients from 2,734 consecutive patients that were referred for an OGD, or consultation and OGD, to a single gastroenterologist in a regional center in Queensland during the period of 01/01/2004-01/04/2009. All data was prospectively entered for later interrogation.

### Inclusion/Exclusion Criterion

Patients were excluded from routine biopsy if they presented with gastrointestinal bleeding, had a coagulopathy or if they had had a previous normal duodenal biopsy within 5 years. Patients previously diagnosed with CD were excluded from this study. 175 adult patients (130 female and 45 male) were excluded under these conditions.

### Intervention

All 2,559 eligible patients had presenting clinical details and celiac antibody results prospectively recorded and underwent a biopsy of the second part of the duodenum as part of their clinical workup. All patients proceeded to OGD and including a duodenal biopsy. All newly diagnosed CD patients had follow up coeliac Ab tests (if not already performed pre-operatively organized by referring doctor) before starting a gluten free diet (GFD).

A positive diagnosis of CD was made if the duodenal histology revealed a Marsh criteria grade IIIa lesion (intra-epithelial lymphocytosis and crypt hyperplasia plus mild shortening of villi) or higher.

### Data collection

CD patients diagnosed over a 5-year period were analysed with respect to age, gender, presenting clinical symptoms, endoscopic appearance and pre- and post- endoscopy celiac Ab status. Patients aged less that 16 years were classified as children. The major presenting clinical symptoms were divided into Major and Minor Clinical Indicators (CI’s) to duodenal biopsy [1],[7]. Major CI’s (symptoms of diarrhea, weight loss and iron deficiency, a positive pre-operative coeliac Ab and a positive family history of CD) are likely to be associated with a greater chance of CD diagnosis and minor CI’s (other gastrointestinal and extra-intestinal symptoms) are likely to be associated with a low chance of CD diagnosis (Table 1)[11].

**Table 1:** Minor Clinical Indicator for Duodenal biopsy.

Non-specific Gastrointestinal Symptoms	Non-specific extra-intestinal symptoms
Constipation Dyspepsia	Fatigue
Abdominal Bloating Flatulence	Arthralgia
Vomiting Nausea	
Abdominal Pain Reflux	
Non-alcoholic steatohepatitis (NASH)	

### Celiac antibody

Celiac Ab tests had been requested by the referring doctor in a many patients pre- operatively. Patients with no pre-operative Ab results proceeded to an Ab test before starting a GFD if the duodenal biopsy showed CD. The celiac Ab tests performed were either an IgA antiendomysial (AEAb) or IgA tissue transglutaminase antibody (tTGAb) test with a comparative total serum IgA level. If the total IgA level was low the patient proceeded to an IgG antigliaden Ab (AGAb) test. The tests were performed by Sullivan and Nicolaides Pathology service (Genesis tTG IgA Eliza kit). The 2013 recommended Medicare fee for this test is AUS$39.90.

### Endoscopy Details

A single gastroenterologist performed all endoscopies in one of two Toowoomba hospitals, both accredited for endoscopy. Olympus endoscopic equipment was used (2004-2009 Olympus Series Q145, not high definition or zoom). The duodenal mucosal appearance was classified as either “High suspicion of CD” (nodular mucosa with mosaic mucosal pattern and scalloping), “Possibility of CD” (subtle mucosal changes with impression of loss of surface texture and reduced duodenal folds) or “Normal”. A minimum of two mucosal biopsies was routinely taken from the second part of the duodenum for histology. Duodenal biopsy specimens were fixed in buffered formalin and embedded in paraffin wax. Sliced 3 mm sections were stained with hematoxylin and eosin and reported routinely by a single pathology service. The 2013 recommended Medicare fee for a duodenal biopsy is AUS$97.

### Statistical Analysis

All data was entered in a database (File maker Pro v6). For tests of significance between two proportions, a 95% confidence interval was constructed around the difference between the proportions, and a chi-squared test of significance performed. For two by two contingency tables with less than 5 observations in any cell, Fisher’s Exact Test was performed. For a test of linear trend regarding proportions of patients presenting with major symptoms in different age groups, a logistic regression analysis was performed. In all cases, a p value of less than 0.05 was considered significant, and all analyses were performed using SPSS v17.

### Ethics

After pre-operative discussion all patients undergoing upper endoscopy signed a consent form to perform the endoscopy and including duodenal biopsy (which was included as routine clinical practice at this institution). Patient anonymity was preserved in the audit of the clinical data and further ethics permission was not sought.

## Results

**Figure 1:**
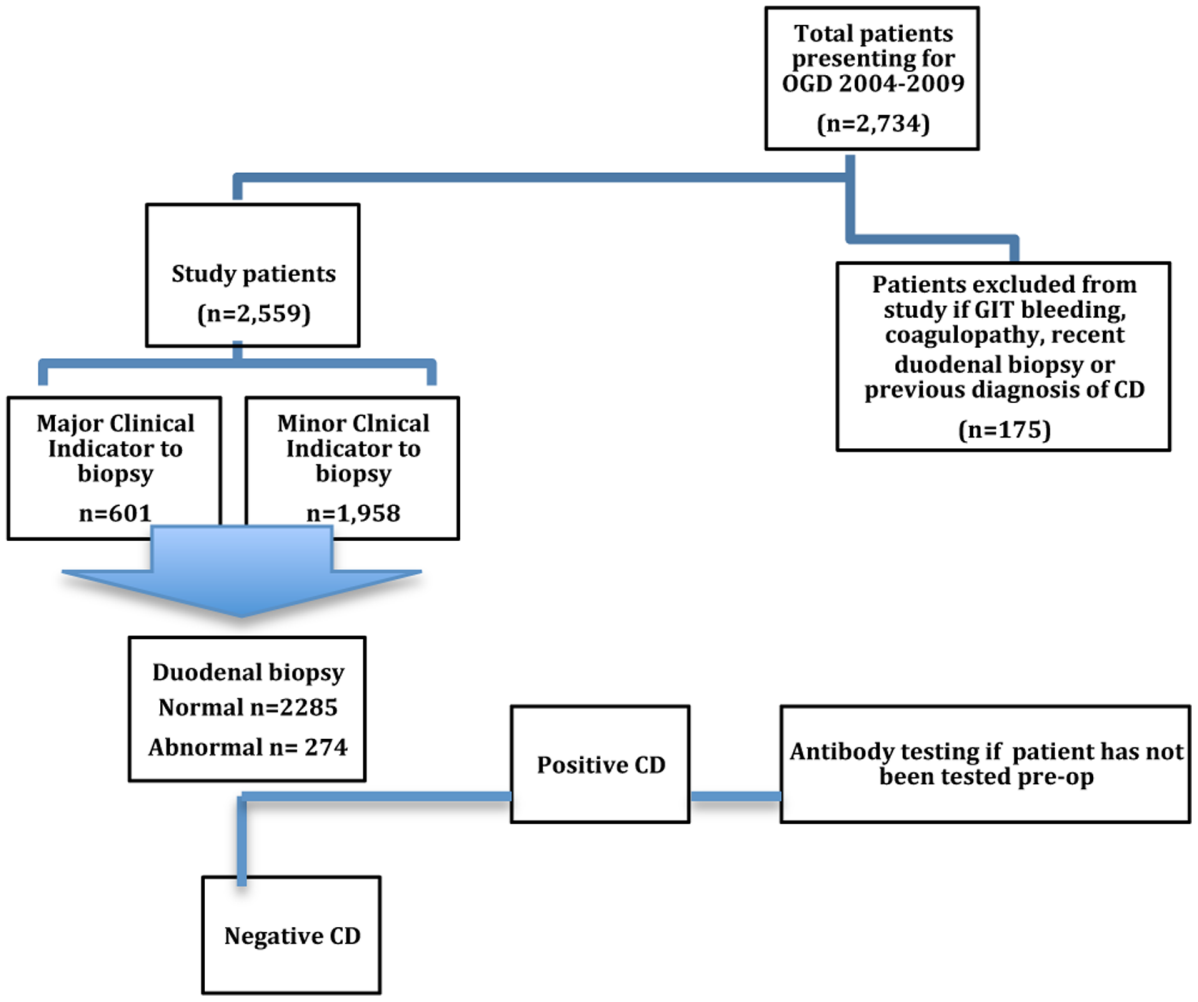
Outline of study.

Of the eligible 2,559 OGD’s performed from 2004–2009, 2,496 were adult patients and the remaining 63 were children. Thirty five patients were newly diagnosed with CD, confirming that the prevalence of newly diagnosed CD in all comers presenting for OGD is high (1.4%) (Figure 1). This is an average of 7 new CD patients per year who are newly diagnosed in this gastroenterology practice. Newly diagnosed adult CD patients in this study numbered 29/2,496 (1.2%): one new adult patient every 8 weeks. No adverse events occurred related to the duodenal biopsy.

### Clinical Presentation

Over three quarters of the total group of OGD patients presented with Minor Clinical Indicators (1,958 patients, 76.5%) The majority of the total CD cohort (27/35; 77%) presented with Major CI’s for Duodenal Biopsy but 8 (23%) patients overall had Minor CI’s for Duodenal Biopsy (Table 2).

**Table 2:** Clinical Indicators among the 35 CD patients.

Major Clinical Indicators					
	Total	Pre-op Ab Positive	Iron deficiency	Family History	Diarrhoea/weight loss
Number of CD patients	27	21	8	5	12

Minor Clinical Indicators					
	Total	Abdominal Pain	Bloating	Reflux symptom s	Nausea/Dyspepsia/ Vomiting/Fatigue/ Hepatitis
Number of CD patients	8	5	1	3	8

All of the 6 children presented with a Major CI to biopsy (2 family history but asymptomatic, 2 iron deficient and 2 with atypical symptoms) and 5/6 had positive coeliac Ab prior to endoscopy.

Eight of the 29 adult new CD patients (28%) presented with Minor CI’s to biopsy (Table 3). Four of the eight patients (50%) aged 60 years and above presented with Minor CI’s to Duodenal Biopsy compared with only 19% of the 16 – 59 year age group. As they age, patients present less commonly with Major CI’s: 100% as children, 81% as adults, and 50% as 60+ (Table 3). On logistic regression, the negative linear trend with age was statistically significant (2-sided p = 0.03).

**Table 3:** Comparison of clinical indication for duodenal biopsy by age.

		Major Criteria	Minor Criteria
**Children**	(*n = *6)	6 (100%)	0
**Adults 16**–**­-59**	(*n* = 21)	17 (81%)	4
**Adults 60+**	(*n* = 8)	4 (50%)	4

### Age at diagnosis

Six of the 35 newly diagnosed CD patients were children (ranging from 2–15 years) at diagnosis. The age of the 29 adults ranged from 23–76 years (median age at diagnosis 47 years) and 8/29 (28%) adult patients were aged 60 years or over at diagnosis.

### Gender

Overall 8/667 males and 27/1829 females had CD, giving gender prevalence rates of 1.2% and 1.5% respectively. The adult CD patients had similar gender prevalence: 6/647 (0.9%) males and 23/1786 (1.3%) females. Although there was a trend for CD to be more common in females this finding did not reach significance in the total cohort, or in different age groups (difference over total cohort of –0.3%: 95% CI –1.3% – 0.8%; p = 0.6). Adult females were more likely to present with Major Clinical Indicators than adult males (78% v 50%, Table 4) but this finding did not reach statistical significance (p = 0.3).

**Table 4:** Comparison of Clinical Indication for duodenal biopsy by age and gender.

Age at diagnosis	Female children	Male children	Females 16–59 yrs	Males 16–59yrs	Females >60 yrs	Males >60yrs	All female adults	All male adults
**Number (%) of new CD**	4 (11%)	2 (6%)	18 (51%)	4 (11%)	5 (14%)	3 (9%)	23 (66%)	6 (17%)
**Major Criteria number**	4	2	15	2	3	1	18	3
**Minor criteria number**	0	0	3	1	2	2	5	3

### Antibody Status

Antibody status was available on all CD patients. One patient with a Major CI was negative for coeliac Ab although he was not IgA deficient, and the remaining patients (34/35) had either pre- or post-endoscopy positive coeliac Ab giving a diagnostic test sensitivity of 97%. Although 3 (9%) patients had total serum IgA below the normal range they all had positive tTGAb levels. All patients with positive tTGAb or AEAb recorded in this study had CD confirmed on biopsy.

### Endoscopic appearance

The duodenal mucosa was abnormal in 274 (10.7%) of OGD patients and 30/274 (10.9%) of these were found to have CD. Severe changes were seen in 18/274 patients and all were diagnosed to have CD. Only 93 of 274 OGD patients with abnormal duodenal mucosa presented with Major Clinical Indicators.

The duodenal mucosa looked definitely atrophic in only 18/35 (51%) of the CD patients. The duodenal mucosa showed mild abnormalities in 12/35 (34%) and looked apparently normal in 5/35 (14%). Patients who presented with Major CI’s had definite endoscopic villous atrophy in 14/27 (52%) of cases; comparable to patients presenting with Minor CI’s (4/8; 50%) (Table 5).

**Table 5:** Endoscopic appearance in CD patients presenting with Major and Minor Clinical Criteria.

		Macroscopic Endoscopic Appearance		
	Total	Highly likely CD	Possible CD	Normal
MAJOR				
No of CD patients	27	14	10	3
Pre-op Ab positive		13	5	3
Total Ab positive		14	10	3
Ab negative		0	1	0
				
MINOR				
No of CD patients	8	4	2	2
Post-op Ab positive		4	2	2
Ab negative		0	0	0

+ Antibody (Ab) for the majority was tTGAb or AEAb, a small number AGAb.

Ab testing always postoperative for Minor criteria, sometimes pre-operative for Major.

Comparison of endoscopic appearance and age at diagnosis showed that 67% of the children, 43% of the 16–59 age group, and 62% of 60+ presented with definite endoscopic villous atrophy.

### Diagnostic Predictive Value

The sensitivity and specificity of Clinical Indicators, endoscopic appearance and coeliac antibody status was assessed. Coeliac antibody status was only available in the patients who presented pre-operatively with this result or in patients subsequently diagnosed on biopsy with CD so that antibody specificity cannot be assessed.

#### Major Clinical Indicator and positive coeliac antibody

All of 35 CD patients were identified by either a positive coeliac Ab or presentation with a Major CI (sensitivity 100%).

#### Abnormal duodenal mucosa and positive celiac antibody

All of the 35 CD patients were identified by either a positive celiac Ab or abnormal duodenal appearance (sensitivity 100%).

#### Positive Coeliac antibody alone

One of the 35 patients did not have a positive Ab (sensitivity  =  97%, 95% confidence interval 91 – 100%).

#### Major Clinical Indicator and/or macroscopically abnormal duodenal mucosa

Overall 782 patients presented with either a Major CI or abnormal duodenal mucosa. The number of new CD patients who presented with a Major CI for biopsy or an abnormal endoscopic appearance was 33/35 (sensitivity 94% with 95% confidence interval of 86 – 100%; specificity 4.2% and 95% confidence interval of 2.8 – 5.6%).

#### Minor Clinical indicator and/or abnormal duodenal mucosa

Eight CD patients presenting for endoscopy with a Minor CI for biopsy represented only 23% of the total CD group but because of the nature of their non-specific symptoms none of these patients had had pre-operative coeliac Ab performed. In this group of low clinical suspicion only 4/8 (50%) had severe duodenal abnormalities that would have routinely indicated that a biopsy was needed.

## Discussion

The prevalence of newly diagnosed CD in our patients presenting for OGD over the last 5 years in our population was 1.4% (1.2% in the adult patients). This is higher than the reported prevalence of CD of 0.5–1.0% in the community [2], [5], [7], [8]. It is not unexpected that CD is more common in this patient cohort considering that patients with CD present with gastrointestinal symptoms for investigation but almost a quarter of these patients presented with non-specific or atypical gastrointestinal symptoms such as reflux symptoms. One quarter of the newly diagnosed adult CD patients in this study were over the age of 60 years at diagnosis. This finding accords with a 1994 UK study that found 19% of patients with newly diagnosed CD were over 60 years and furthermore one third of these patients had attended family doctors and hospital outpatients for an average of 28 years before the diagnosis was made [13].

Our study confirms previous findings [13], [15], [16] that the clinical presentation of CD changes across the life span. As people age they present progressively less commonly with Major CIs. This finding will be relevant for clinicians seeing older adults complaining of non-specific gastrointestinal symptoms. Patients presenting with a more classical presentation are more easily diagnosed at an earlier age than those with a more atypical picture for whom diagnosis remains elusive for longer. However perhaps the onset of celiac disease in older adults is associated with a genuinely different symptom complex. At the other end of the age spectrum, the pediatric population presented solely with Major CI’s. Children with CD may present more commonly with classical symptoms although it is possible that CD children with Minor CI’s may not be considered unwell enough to warrant investigations/endoscopy and remain undiagnosed till adulthood.

The ready availability and general awareness of CD Ab testing ensured that 75% of these newly diagnosed CD patients had had a positive CD Ab test prior to the OGD. The remaining 25% of the patients may not necessarily have been diagnosed as many endoscopists in routine clinical practice rely on the endoscopic appearance alone to trigger a decision to biopsy the duodenum even if the patient has presented with Major CI’s such as diarrhoea, weight loss or iron deficiency [3]. Current international guidelines recommend 4 orientated biopsies of the duodenum (one from the bulb and three from the second part). In 2008 Pais et al [17] demonstrated an increase in diagnostic accuracy with 4 compared to 2 biopsies (100% versus 90%). Our study started in 2004 and to be consistent only 2 biopsies were taken during the 5 years of the study. It is possible had more biopsies been taken our diagnostic yield may have been approximately 10% higher. Half of the patients presenting with atypical symptoms in this study had not had pre-operative coeliac Abs performed, had non-specific endoscopic appearances and without biopsy 11% of new CD cases would have been missed.

Severe duodenal atrophic changes are recognized by the experienced endoscopist and are reported to have a high specificity for CD [17], confirmed in this study. Appreciation of subtle changes in the appearance of the duodenum has been reported to be relative to the level of skill of the endoscopist but in this dedicated study almost half of the CD patients had either subtle changes (34%) or normal looking (14%) duodenum. There was no relationship between definite endoscopic mucosal abnormalities and clinical presentation or age at diagnosis in our study. It is well recorded in the literature that sole reliance on the macroscopic duodenalm appearance leads to the detection of CD in only 50–87.5% of cases [6]. The endoscopic appearance of the mucosa is considered a relatively poor diagnostic tool.

Our study revealed that Coeliac Ab tests were positive in 97% of patients subsequently diagnosed to have CD confirmed on duodenal biopsy. If we were to biopsy only Coeliac Ab positive and Major CI cases 100% of cases of CD were identified in our cohort. Our figures closely resemble those reported in a 2007 UK study [1] that concluded that performing routine Ab tests (tTGAb) in combination with biopsy of all cases presenting with high-risk symptoms (anaemia, weight loss, diarrhoea - our “Major CI’s”) resulted in the detection of all cases of CD. Assessing the duodenal mucosa is subjective but we also found 100% of CD patients were identified if we biopsied all positive antibody patients and those with abnormal mucosa. Missing any patient with CD is undesirable at the least, with the long-term complications of undiagnosed symptomatic CD leading to reduced quality of life, increased morbidity, and mortality [5], [10].

Histologic examination of the duodenum is the gold standard for diagnosing CD. However routine biopsy of the duodenum is expensive. An Australian study [18] assessed the cost-effectiveness of random small bowel biopsy in patients presenting with iron deficiency. Four new cases of CD were found from 253 biopsies taken (2% prevalence) ($72.15/biopsy), equating to $4563.49 per new case. This figure could be reduced to $2435 when only those less than 60 years of age were tested. No case of CD would be missed in their experience by the lower age threshold, although our study results suggest 28% of the adult CD cases would have been missed using that age cut-off. The cost of CD antibody testing in this study [18] was only $25.00 per sample, significantly cheaper than the cost of biopsy assessment.

If we assume a relatively high false positive rate (10%) of CD antibody testing [19] then we could have expected 39 patients in our total OGD group to be positive to antibody testing. Using current 2013 costing we can calculate that to test all OGD patients in this study for celiac antibody would cost AUS$102,360. If we then biopsy the positive Ab patients, positive Ab and Major CI patients and positive Ab and abnormal duodenal mucosa patients the cost to diagnose one new CD patient in this study would be AUS$3,027, $4,629 and AUS$3,710 respectively. The development of cheaper or more specific antibody kits and the detection of more new cases of coeliac disease if four duodenal biopsies had been taken would see these costs reduce in the future.

The majority of patients in our study presented to the referring doctor with symptoms, mostly gastrointestinal. The exceptions were five asymptomatic patients who presented for screening OGD. Three had a family history of CD and hence were tested for coeliac Ab, with all found to be positive. These patients may not have been entirely asymptomatic as it is often the family members who harbour doubts about their overall health who proceed to follow-up. The other two patients were undergoing screening in relation to their primary diagnoses of Type One Diabetes in one patient and Osteoporosis in the other. There have been a number of studies that suggest that screening the general population for CD is not cost effective [5], [20]–[22] and may actually decrease quality of life associated with imposing a GFD on asymptomatic patients [5]. However it is important to differentiate screening of the general population from adequate investigation of patients presenting with symptoms that have prompted further medical investigation in the form of an OGD.

In conclusion, 10% of patients with CD may be missed at routine endoscopy if indication to biopsy the duodenum is predicated upon endoscopic appearance or classic clinical presentation. A pre-operative coeliac antibody test, in conjunction with clinical history and careful endoscopic evaluation, may provide a cost-effective clinical algorithm that will improve the diagnostic yield of symptomatic coeliac patients presenting for an upper endoscopy.
